# Exploring CAR cell therapies beyond CAR-T for myeloid malignancies

**DOI:** 10.1186/s12929-026-01265-8

**Published:** 2026-06-04

**Authors:** Yan-Ruide Li, Yuning Chen, Lili Yang

**Affiliations:** 1 Department of Microbiology, Immunology and Molecular Genetics, University of California, Los Angeles, Los Angeles, CA 90095 USA; 2 Department of Bioengineering, University of California, Los Angeles, Los Angeles, CA 90095 USA; 3 Molecular Biology Institute, University of California, Los Angeles, CA 90095 USA; 4 Eli and Edythe Broad Center of Regenerative Medicine and Stem Cell Research, University of California, Los Angeles, Los Angeles, CA 90095 USA; 5 Jonsson Comprehensive Cancer Center, David Geffen School of Medicine, University of California, Los Angeles, Los Angeles, CA 90095 USA; 6 Parker Institute for Cancer Immunotherapy, University of California, Los Angeles, Los Angeles, CA 90095 USA; 7 Goodman-Luskin Microbiome Center, University of California, Los Angeles, Los Angeles, CA 90095 USA

**Keywords:** Myeloid malignancies, Acute myeloid leukemia (AML), Myelodysplastic syndromes (MDS), Chimeric antigen receptor (CAR), CAR-T, CAR-NK, CAR-NKT, CAR-macrophage, Bone marrow homing, On-target off-tumor effect, Leukemia stem cells (LSCs)

## Abstract

Chimeric antigen receptor (CAR)-T cells have demonstrated remarkable efficacy in several hematologic malignancies; however, their application in myeloid malignancies such as acute myeloid leukemia (AML) and myelodysplastic syndromes (MDS) remains limited, with no FDA-approved products to date. This limited progress largely reflects both efficacy challenges and safety concerns. CAR-T cells demonstrate poor trafficking and persistence within the bone marrow, limited activity against leukemia stem cells (LSCs), and reliance on antigens such as CD33 and CD123 that are also expressed on normal hematopoietic stem and progenitor cells, resulting in significant on-target off-tumor toxicity. Given these limitations, attention has increasingly shifted toward alternative CAR-engineered immune cells, including CAR-natural killer (CAR-NK) cells, CAR-invariant natural killer T (CAR-NKT) cells, and CAR-macrophages (CAR-Ms). These platforms offer unique advantages, such as intrinsic antitumor activity, distinct trafficking properties, reduced risk of graft-versus-host disease (GvHD), and potentially safer antigen recognition profiles, that may help overcome barriers faced by CAR-T cells. In this review, we highlight the challenges of applying conventional CAR-T cells to myeloid malignancies, examine emerging alternative CAR-cell platforms, and discuss how their unique biology and engineering strategies may provide safer, more effective, and more accessible therapeutic options for patients with these difficult-to-treat cancers.

## Introduction

Myeloid malignancies, including acute myeloid leukemia (AML) and myelodysplastic syndromes (MDS), originate in the bone marrow (BM) and disrupt normal hematopoiesis [1–4]. Standard treatment typically consists of intensive chemotherapy with agents such as daunorubicin and cytarabine, followed by allogeneic stem cell transplantation (allo-HSCT). In recent years, additional therapeutic options, including hypomethylating agents (HMAs), monoclonal antibodies, antibody–drug conjugates (ADCs), and targeted inhibitors, have expanded the treatment landscape [5–8]. Nevertheless, long-term outcomes remain poor, with 5-year survival rates of only ~ 37% for AML and ~ 30% for MDS [1–4]. These therapies are often limited by resistance and disease relapse, driven in part by the persistence of malignant blast cells within the BM. In particular, leukemia stem cells (LSCs), defined by their self-renewal capacity and ability to sustain disease propagation, represent a major barrier to durable remission [9–12]. Thus, there is an urgent need for innovative therapeutic strategies that can effectively target BM-resident LSCs and address the substantial unmet clinical needs of patients with myeloid malignancies.

Chimeric antigen receptor (CAR)-T cell therapy has emerged as a promising immunotherapeutic approach for myeloid malignancies [6, 13, 14]. CARs are synthetic, genetically engineered receptors that redirect T cells to recognize specific tumor-associated antigens, thereby enhancing T cell activation and cytotoxicity against malignant cells. In AML and MDS, several candidate antigens have been explored as CAR targets, including CD33, CD123, CD7, CD70, Fms-like tyrosine kinase 3 (FLT3), and C-type lectin-like molecule-1 (CLL1) [14–17]. Preclinical studies and early-phase clinical trials have provided valuable insights into the feasibility, safety, and preliminary efficacy of CAR-T cell therapy in these diseases.

**Fig. 1:**
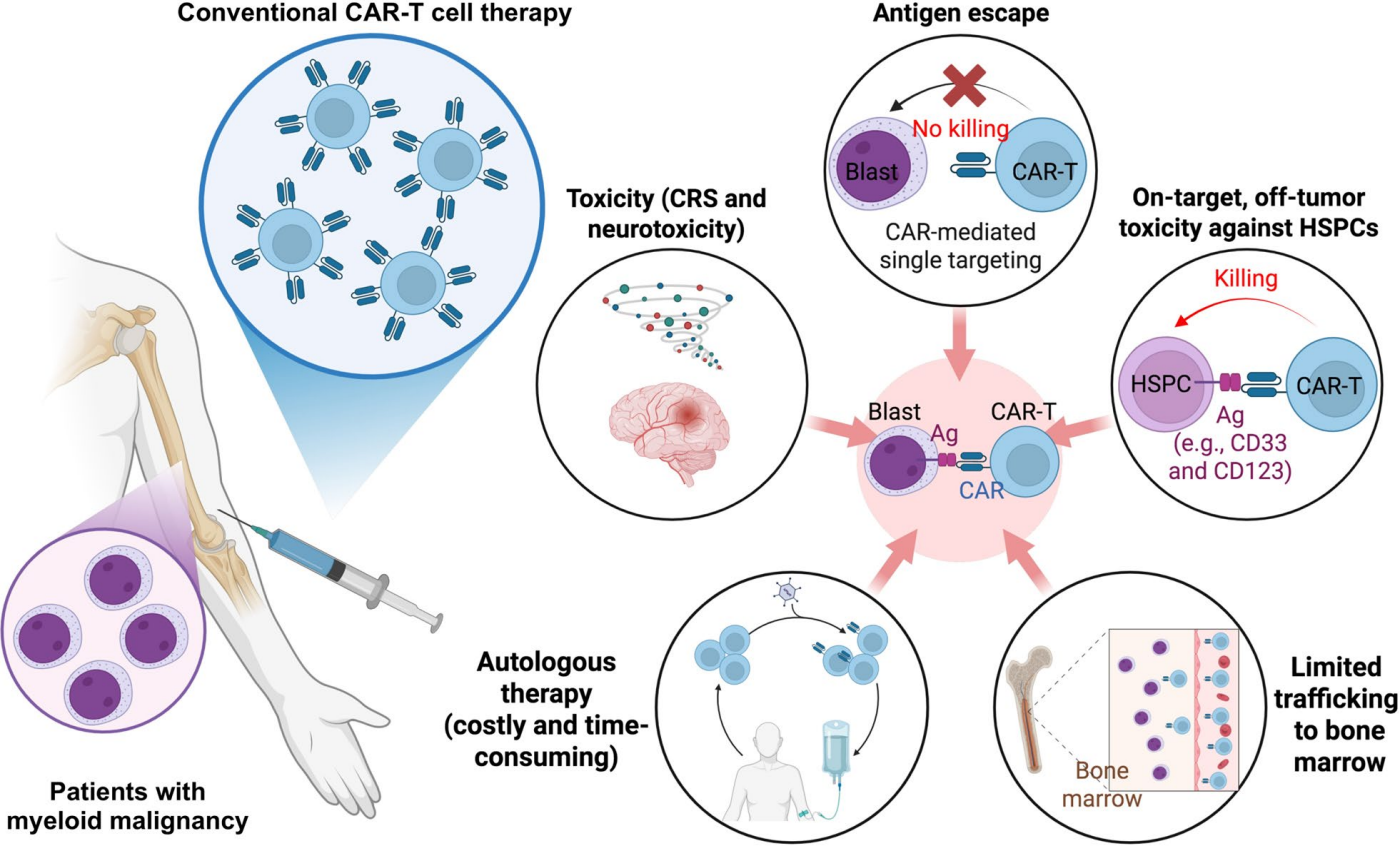
Limitations of conventional CAR-T cell therapy in myeloid malignancies. Conventional CAR-T cell therapy for myeloid malignancies faces significant challenges that limit its clinical success. A major issue is the lack of ideal target antigens, as commonly targeted markers like CD33 and CD123 are also expressed on normal hematopoietic stem and progenitor cells, leading to severe on-target, off-tumor toxicity and prolonged bone marrow suppression. Additionally, CAR-T cells often show limited trafficking to the bone marrow and struggle to eliminate leukemia stem cells, which are crucial drivers of relapse. The immunosuppressive bone marrow microenvironment further impairs CAR-T function and persistence, while antigen heterogeneity and loss enable leukemia escape. Patients are also at high risk for cytokine release syndrome, neurotoxicity, and myeloablation. Finally, manufacturing challenges, particularly in older or heavily pretreated patients, and the potential for graft-versus-host disease in allogeneic approaches, further constrain efficacy and broader application. These limitations underscore the need for alternative cellular platforms

Despite these advances, the clinical outcomes of CAR-T cell therapy in AML and MDS remain less encouraging compared to its success in CD19-positive B cell malignancies and BCMA-positive multiple myeloma (MM). Several factors contribute to these limitations (Fig. 1). First, CAR-T cells demonstrate poor trafficking to and limited persistence within the bone marrow microenvironment, resulting in suboptimal tumor infiltration and engagement [18]. Second, LSCs and other malignant subpopulations can evade immune surveillance by downregulating target antigens, rendering them resistant to CAR-mediated cytotoxicity [19, 20]. Third, many candidate antigens, including CD33 and CD123, are expressed not only on malignant cells but also on normal hematopoietic stem and progenitor cells (HSPCs), leading to substantial on-target, off-tumor toxicity and prolonged myeloablation [14, 21–23]. In addition, the majority of current CAR-T cell therapies are autologous, which limits their broad applicability due to manufacturing complexity, variability in patient T cell quality, and the risk of graft-versus-host disease (GvHD) when considering allogeneic transfer [24–28]. Consequently, there is an urgent need for next-generation cell therapies that can overcome these limitations, offering off-the-shelf availability, reduced toxicity, and improved efficacy in targeting malignant cells while sparing normal hematopoietic compartments [29].

Alternative CAR-engineered immune cells are being actively explored to overcome the limitations of conventional CAR-T therapies. CAR-natural killer (CAR-NK) cells harness the innate cytotoxicity and cytokine-secreting capabilities of NK cells, providing potent antitumor activity with a lower risk of GvHD, which makes them suitable for allogeneic, off-the-shelf applications [30–33]. Similarly, CAR-invariant natural killer T (CAR-NKT) cells combine the tumor-targeting specificity of CARs with the unique immunoregulatory properties of NKT cells, enabling efficient trafficking to tumor sites, modulation of the tumor microenvironment (TME), and reduced alloreactivity [32, 34–37]. In addition, CAR-macrophages (CAR-Ms) are engineered to enhance phagocytosis of tumor cells, remodel the immunosuppressive TME, and promote recruitment and activation of adaptive immune responses [38–41]. Collectively, these next-generation CAR-engineered immune cells offer versatile platforms to address key limitations of CAR-T therapies, including limited persistence, inadequate tumor infiltration, and on-target off-tumor toxicity.

In this review, we provide a comprehensive overview of the current preclinical development of alternative CAR-engineered immune cells, with a particular focus on their advantages and limitations in the treatment of myeloid malignancies. We also examine the emerging clinical landscape, highlighting early-phase trials evaluating these therapies, especially CAR-NK cells. Finally, we discuss future directions for the field, aiming to provide a thorough perspective on strategies to optimize efficacy, safety, and broad applicability of next-generation CAR cell therapies. To provide a conceptual framework for comparing these platforms, we organize this review around the major barriers that have limited conventional CAR-T therapy in AML and MDS, including inadequate trafficking to the bone marrow, insufficient activity against heterogeneous leukemia stem and progenitor cell populations, antigen overlap with normal hematopoietic stem and progenitor cells, limited persistence or excessive persistence-associated toxicity, and the difficulty of developing scalable allogeneic products. Within this framework, CAR-NK, CAR-NKT, and CAR-M platforms should not be viewed as interchangeable alternatives, but rather as biologically distinct therapeutic systems.

### Preclinical studies of CAR cell therapies beyond CAR-T for treating myeloid malignancies

#### CAR-NK cells

**Fig. 2:**
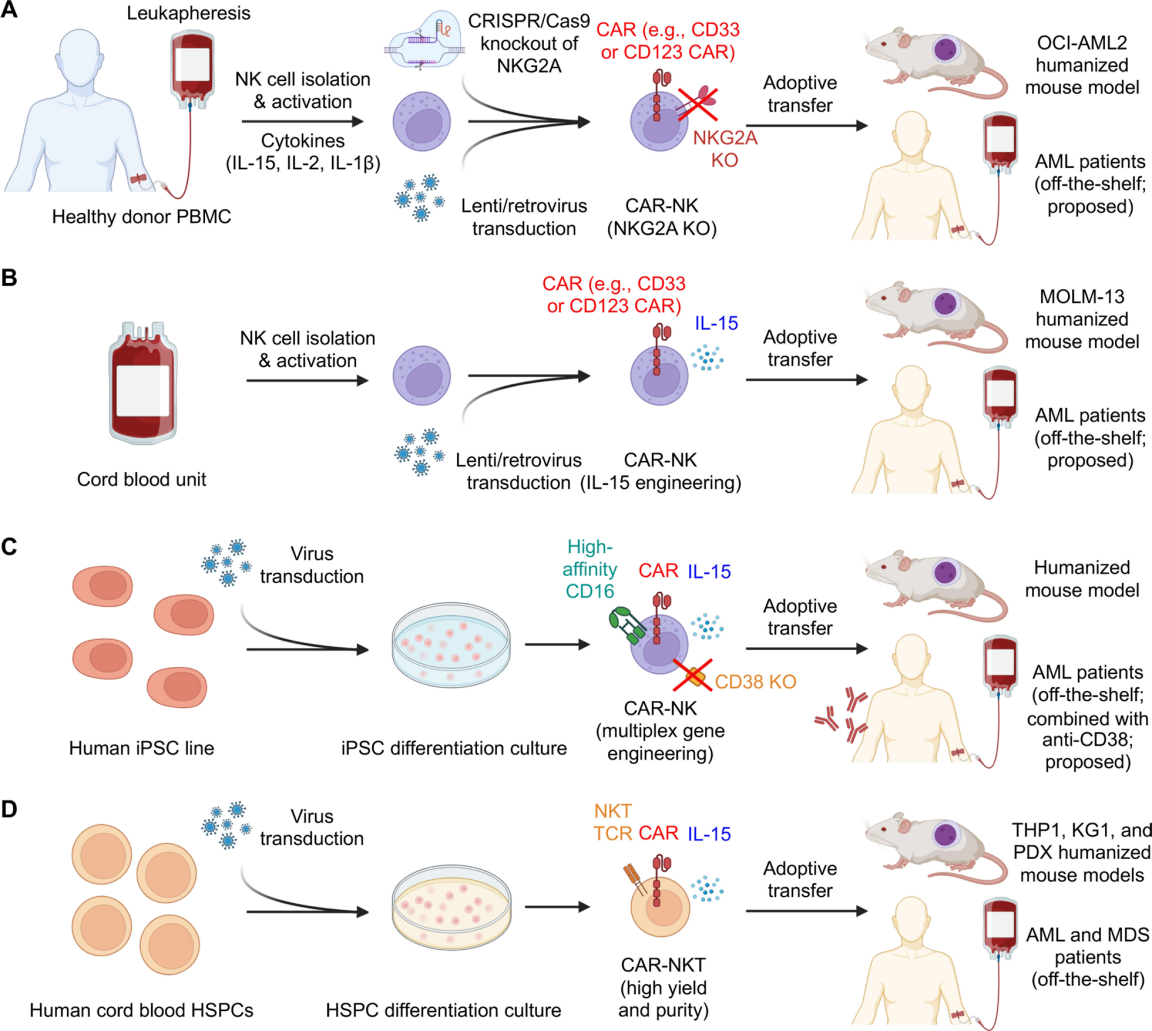
Development of alternative CAR cells in treating myeloid malignancies. Key examples of CAR-NK and CAR-NKT cell generation for treating myeloid malignancies include producing CAR-NK cells from healthy donor peripheral blood (**A**) [49], cord blood (**B**) [60], or via iPSC differentiation (**C**) [66], as well as generating CAR-NKT cells from HSPCs using a clinically guided culture method (**D**) [18]

NK cells are critical innate immune effectors that provide rapid responses against virally infected and malignant cells. Unlike T cells, whose activation is antigen-specific and MHC-restricted, NK cells rely on a balance of signals from activating and inhibitory receptors to recognize and eliminate abnormal cells. Key activating receptors on NK cells include NKG2D, NKp30, NKp46, 2B4, and CD16, which detect stress-induced ligands or antibody-coated targets on potential threats. These receptors associate with adaptor molecules such as DAP10, CD3ζ, and FcεRIγ to initiate robust cytotoxic responses through degranulation and cytokine secretion [30, 42–44]. The unique biology of NK cells offers distinct advantages for cellular immunotherapy. Importantly, allogeneic NK cells do not mediate GvHD, although they may release inflammatory cytokines that can amplify pre-existing tissue pathology. This immunological profile allows NK cells to be developed as “off-the-shelf” therapeutics that can be manufactured in large batches and stored for rapid clinical use, overcoming key limitations associated with patient-specific CAR-T therapies, including high production costs and treatment delays [30, 32, 45, 46]. Moreover, CAR-NK cells can be designed to combine innate cytotoxicity with antigen-specific targeting, enhancing their potency against myeloid malignancies while potentially reducing the risk of on-target, off-tumor toxicity. Preclinical studies have demonstrated that CAR-NK cells exhibit robust anti-leukemic activity, maintain favorable safety profiles, and can be further optimized for persistence and in vivo expansion using cytokine support or genetic modifications (Table 1 and Fig. 2).

**Table 1:** Preclinical exploration of CAR cell therapies beyond CAR-T for myeloid malignancies

CAR cell type	Disease indication	CAR target	Additional genetic engineering	CAR cell manufacturing	Efficacy result	Safety result	Year and References
CAR-NK	AML	CD123	IL-15 engineering	Retrovirus-based transduction of primary NK cells isolated from peripheral blood of healthy donors	2B4.ζ CAR-NK cells demonstrate superior anti-AML activity compared with NK cells and 4-1BB.ζ CAR-NK cells in MV-4–11 and MOLM-13 xenograft mouse models	Constitutive IL-15 expression causes lethal toxicity	2021 [50, 142]
CAR-NK	AML	NKG2D ligand	IL-15 engineering	Non-viral piggyBac transposon transduction of primary NK cells isolated from peripheral blood of healthy donors	IL-15 enhances the in vitro and in vivo persistence ofs CAR-NK cells, resulting in improved tumor control and prolonged survival in the KG-1 AML model	Not report	2021 [58]
CAR-NK	AML	CD33	High-affinity, non-cleavable CD16 (hnCD16) engineering; IL-15 engineering; CD38 knockout	iPSC differentiation into allogeneic CAR-NK cells	CAR-NK cells exhibit potent anti-tumor activity against multiple AML cell lines and patient-derived blasts, independent of NKG2D ligand expression levels; show superior ADCC when administered with daratumumab	Not report	2022 [66]
CAR-NK	AML	CD123	None	Retrovirus-based transduction of primary NK cells isolated from peripheral blood of healthy donors	CAR-NK cells show significant anti-leukemia activity in vitro and in THP1 xenograft mouse models	Superior safety with minimal on-target/off-tumor effects in an HSC-engrafted humanized mouse model compared with CAR-T cells	2022 [54]
CAR-NK	AML	CD33	None	Baboon envelope pseudotyped lentivirus-based transduction of primary NK cells isolated from peripheral blood of healthy donors	CAR-NK cells reduce leukemic burden and prevent bone marrow engraftment of leukemic cells in OCI-AML2 xenograft mouse models	No clinical signs of CRS, weight reduction, altered appearance or behavior, or GvHD	2022 [47]
CAR-NK	AML	Neoepitope derived from the cytosolic oncogenic NPM1-mutated protein presented by HLA-A2	IL-15 engineering	Lentivirus-based transduction of primary NK cells isolated from peripheral blood of healthy donors	CAR-NK cells exhibit enhanced activity against NPM1-mutated AML cell lines and patient-derived blasts, with sustained in vivo persistence and significant improvement of AML outcomes in xenograft models	No off-target toxicity	2022 [59]
CAR-NK	AML	CD33	Anti-CD16 antibody (B16) engineering	Retrovirus-based transduction of primary NK cells isolated from peripheral blood of healthy donors	Bifunctional CD33/B16 CAR-NK cells show superior killing efficiency toward AML cells compared to CD33 CAR-NK cells	No safety change with additional expression of B16	2023 [55]
CAR-NK	AML	CD33	None	Automated generation of high numbers of CAR-NK cells from healthy donor blood using the CliniMACS Prodigy platform	CAR-NK cells markedly reduce leukemic burden in an OCI-AML2 NSG-SGM3 xenograft model compared with small-scale–produced CAR-NK cells	Not report	2024 [48]
CAR-NK	AML	CD33	*KLRC1* (NKG2A) knockout	Lentivirus-based transduction of primary NK cells isolated from peripheral blood of healthy donors	KLRC1-knockout CAR-NK cells exhibit enhanced cytotoxicity against AML blasts while maintaining an activated state and showing reduced cell cycle activity compared with conventional CAR-NK cells	Not report	2024 [49]
CAR-NK	AML	CLL-1	IL-2 engineering	Not report	Long-flexible CLL1 CAR-NK cells outperform NK controls in cytotoxicity and proliferation against multiple AML cell lines and primary samples; IL-2 engineering outperforms IL-15 engineering in vivo	No toxicity against allogeneic HPSCs	2024 [57]
CAR-NK	AML	CD33 and CD70	None	Retrovirus-based transduction of primary NK cells isolated from peripheral blood of healthy donors	CAR-NK cells synergize with proteasome inhibition in targeting AML	No toxicity against HSCs and PBMCs	2024 [56]
CAR-NK	AML	CD33	IL-15 engineering	Lenti/retrovirus-based transduction of primary NK cells isolated from umbilical cord	CAR-NK cells demonstrate potent in vivo antitumor efficacy in MOLM-13 xenograft mouse models	Significant toxicity to HSCs in the hematopoietic toxicity assay	2025 [60]
CAR-NKT	AML and MDS	CD33	IL-15 engineering	HSPC differentiation into allogeneic CAR-NKT cells using a clinically guided culture method	CAR-NKT cells migrate to the bone marrow and target bone marrow-resident cancer stem cells through CAR, TCR, or NKR mechanisms, demonstrating high tumor-killing efficacy using THP-1, HL-60, KG-1, and PDX xenograft mouse models	No GvHD, low CRS, low long-term toxicity, low on-target off-tumor effect against hematopoietic precursors	2025 [18]

Albinger et al. developed CD33-targeting CAR-NK cells derived from healthy donor–isolated NK cells using baboon envelope–pseudotyped lentiviral vectors (BaEV-LVs) [47]. These CAR-NK cells demonstrated potent anti-leukemic activity, effectively reducing leukemic burden and preventing bone marrow engraftment of OCI-AML2 cells in xenograft mouse models. Importantly, no observable toxicities were reported, cytokine release syndrome (CRS), weight loss, changes in appearance or behavior, or GvHD, consistent with histopathological analyses of the lung, liver, and colon [47]. Building on this work, the same group developed a GMP-compatible, automated platform for large-scale generation of CD33-specific CAR-NK cells using the CliniMACS Prodigy system. CAR-NK cells produced at this scale retained comparable phenotype and cytotoxic function to their small-scale counterparts, supporting the feasibility of clinical translation and the potential application of this platform to a broader range of targets [48].

Subsequently, the same team further addressed the inhibitory effects of the HLA-E–NKG2A immune checkpoint on CAR-NK cells (Fig. 2A) [49]. By combining CD33-specific CAR expression with CRISPR/Cas9-mediated knockout of the NKG2A-encoding *KLRC1* gene, they generated NKG2A-deficient CAR-NK cells [49]. These modified cells exhibited preserved transcriptional features of activation and maturation even after exposure to AML cells, suggesting that disruption of NKG2A can enhance CAR-NK cell function by overcoming immune checkpoint–mediated suppression. Collectively, these studies highlight the potential of CD33-targeted, gene-edited CAR-NK cells as a promising off-the-shelf therapeutic approach for AML, combining potent anti-leukemic activity with an improved safety profile.

Christodoulou et al. generated CD123-targeting CAR-NK cells from healthy donor–derived NK cells and systematically evaluated the impact of different CAR signaling domain designs [50]. CAR constructs incorporating 2B4.ζ or 4-1BB.ζ signaling domains exhibited higher surface expression and enhanced in vitro anti-AML activity compared with alternative designs. In initial in vivo studies, only 2B4.ζ CAR-NK cells demonstrated improved anti-leukemic activity relative to untransduced (UTD) NK cells and 4-1BB.ζ CAR-NK cells [50]. However, this therapeutic benefit was transient, primarily due to limited persistence of CAR-NK cells in vivo. This study also engineered CAR-NK cells to express IL-15, which improved in vivo expansion and antitumor efficacy [50]. Notably, IL-15 expression also resulted in increased systemic toxicity, consistent with prior reports describing IL-15–associated adverse effects in preclinical models and cancer patients [51–53]. These findings underscore both the potential and the challenges of optimizing CAR-NK cell design to balance efficacy and safety in AML therapy.

Several additional preclinical studies have further highlighted the therapeutic potential of CAR-NK cells targeting other CAR antigens for myeloid malignancies. In one study, CD123-targeting CAR-NK cells demonstrated robust anti-leukemic activity in vitro against both CD123⁺ AML cell lines and primary patient-derived blasts [54]. Their efficacy was also confirmed in two xenograft models of human AML using immunodeficient mice. Importantly, compared with conventional CAR-T cells, CAR-NK cells exhibited a markedly improved safety profile [54]. Mice treated with CD123-specific CAR-T cells experienced severe toxicity, with all animals succumbing by day 5 due to on-target, off-tumor effects on human bone marrow, including depletion of total human CD45⁺ cells and human CD34⁺CD38⁻ hematopoietic stem cells. In contrast, CAR-NK cell therapy was well tolerated, with no observed toxicity and all treated animals surviving to the study endpoint. Furthermore, these CAR-NK cells demonstrated minimal endothelial toxicity relative to conventional CAR-T cells, underscoring their favorable safety profile [54].

Zhang et al. advanced CAR-NK design by engineering CD33-targeting cells to concurrently secrete an anti-CD16 antibody (B16) [55]. This bifunctional approach combined direct AML targeting via CD33 with enhanced NK cell-mediated cytotoxicity through Fc receptor engagement by the secreted antibody. Compared with conventional CD33-targeting CAR-NK cells, the CD33/B16 dual-function CAR-NK cells exhibited superior killing efficiency against AML cells, demonstrating the potential of combinatorial strategies to amplify therapeutic efficacy [55]. Sedloev et al. explored combination therapy strategies, generating CD33- and CD70-targeting CAR-NK cells and evaluating their activity in conjunction with proteasome inhibitors [56]. Treatment of AML cells with bortezomib or carfilzomib reduced surface class I HLA expression in a dose- and time-dependent manner, while upregulating stress-associated proteins both transcriptionally and at the cell surface. This modulation of tumor immunogenicity rendered AML cells more susceptible to CAR-NK cell–mediated killing, highlighting the potential of combination approaches to enhance the effectiveness of CAR-NK therapies [56].

Beyond CD33 and CD123, several other target antigens have been explored for CAR-NK cell therapy in AML. CLL-1 represents a particularly promising target due to its high expression on leukemic cells and absence on normal HSPCs [57]. Long-flexible CLL-1 CAR-NK cells demonstrated superior cytotoxicity and proliferation compared with untransduced NK cells across a panel of AML cell lines and primary patient samples, while exhibiting no toxicity toward allogeneic HSPCs in standard colony-forming unit (CFU) assays [57]. Interestingly, in this study, CAR-NK cells engineered with IL-2 outperformed IL-15-expressing counterparts, achieving a 72% reduction in tumor burden, highlighting the potential of structurally optimized, allogeneic CLL-1 CAR-NK cells as off-the-shelf AML therapeutics [57].

Du et al. developed NKG2D ligand–targeting CAR-NK cells using a non-viral piggyBac transposon system for gene delivery, circumventing the need for conventional lentiviral or retroviral transduction [58]. These CAR-NK cells were co-engineered to express IL-15, which significantly enhanced their antileukemic activity in vitro and in vivo. This study illustrates both the therapeutic benefit of IL-15 expression and the feasibility of using piggyBac-based non-viral platforms for cost-effective CAR-NK cell manufacturing [58].

Dong et al. engineered blood-derived NK cells with a TCR-like CAR specific for a neoepitope generated by the NPM1 mutation presented on HLA-A2 [59]. To further enhance functionality, these cells were cultured in the presence of IL-12, IL-15, and IL-18 to induce a cytokine-induced memory-like (CIML) NK phenotype. CAR CIML NK cells exhibited potent cytotoxicity against NPM1-mutated AML cell lines and patient-derived blasts, persisted in vivo, and significantly improved AML outcomes in xenograft models [59]. This approach demonstrates that intracellular, mutation-specific neoantigens can be targeted effectively using CAR-engineered CIML NK cells, supporting future clinical evaluation in HLA-A2⁺ AML patients harboring NPM1c mutations [59].

In addition to peripheral blood NK cells, umbilical cord-derived NK cells have also been employed for CAR engineering (Fig. 2B). Huang et al. generated CD33-targeting CAR-NK cells from cord blood NK cells, which demonstrated efficacy in Molm13 xenograft models [60]. However, these cells exhibited substantial hematopoietic toxicity, reducing colony formation in multiple HSPC lineages, highlighting the importance of source selection and toxicity evaluation for CAR-NK cell development [60].

iPSC-derived NK cells have emerged as a promising platform for off-the-shelf CAR-NK therapies [61–65]. Want et al. reported multiplexed, iPSC-derived CD33 CAR-NK cells (QN-023a) engineered with four genetic modifications: a CD33-specific CAR optimized for NK cell biology, a high-affinity non-cleavable CD16 (hnCD16) to enhance antibody-dependent cellular cytotoxicity (ADCC), IL-15 to improve persistence, and CD38 knockout to prevent fratricide and synergize with daratumumab (Fig. 2C) [66]. These iPSC-derived CAR-NK cells exhibited potent anti-leukemic activity against diverse AML cell lines and primary patient blasts regardless of NKG2D ligand expression. When combined with daratumumab, QN-023a demonstrated enhanced ADCC compared with unmodified NK cells, highlighting its potential as a clinically translatable, off-the-shelf therapy for AML [66].

Across these studies, several engineering principles emerge. First, cytokine support appears to be one of the most effective strategies for improving CAR-NK cell expansion and antileukemic activity, but it also introduces an important safety tradeoff. IL-15 or IL-2 expression can enhance NK cell survival, proliferation, and cytotoxicity. For example, IL-15-secreting CD123 CAR-NK cells showed improved anti-AML functionality but were also associated with systemic toxicities in xenograft models, while IL-2-armored CLL-1 CAR-NK cells demonstrated enhanced in vivo antileukemic activity in preclinical AML models [50, 57]. However, constitutive cytokine secretion may increase the risk of systemic inflammation, autonomous expansion, or cytokine-associated toxicity. Therefore, cytokine engineering is likely to be most clinically feasible when paired with dose control, regulated expression systems, or safety-switch strategies [50, 67]. Second, checkpoint disruption, particularly targeting the HLA-E-NKG2A axis, provides a rational way to improve CAR-NK function in the suppressive AML bone marrow niche without relying solely on cytokine-driven expansion [68, 69]. Third, bifunctional or combination designs, such as CAR-NK cells secreting antibodies or used together with proteasome inhibitors, may enhance tumor susceptibility to NK-mediated killing, but they also increase manufacturing and regulatory complexity. In support of this concept, proteasome inhibitor pretreatment with bortezomib or carfilzomib enhanced NK cell activity against AML cells and further improved the antileukemic efficacy of CD33- and CD70-specific CAR-NK cells [56]. Finally, TCR-like CAR approaches targeting intracellular mutation-derived neoepitopes, such as NPM1c/HLA-A2, may improve specificity and reduce hematopoietic toxicity, but their clinical applicability will be restricted to patients with the relevant mutation and HLA background. In one study, cytokine-induced memory-like NK cells expressing an NPM1c/HLA-A2-specific TCR-like CAR showed potent activity against NPM1-mutated AML cell lines and patient-derived blasts, persisted in vivo, and improved outcomes in xenograft models [59]. Thus, the most promising CAR-NK strategies for translation are likely those that balance improved persistence and potency with controllable safety, scalable manufacturing, and clear patient-selection criteria.

Overall, preclinical studies of CAR-NK cells for the treatment of myeloid malignancies demonstrate substantial promise as a safe and effective alternative to conventional CAR-T cel therapies. CAR-NK cells combine innate cytotoxicity with antigen-specific targeting, enabling potent anti-leukemic activity while minimizing risks of GvHD and severe systemic toxicities. Target antigens such as CD33, CD123, CLL-1, and NKG2D ligands have been successfully exploited, with engineered CAR-NK cells demonstrating robust cytotoxicity against AML cell lines and patient-derived blasts, as well as efficacy in multiple xenograft models (Table 1). Innovative strategies, including checkpoint blockade via NKG2A disruption, co-expression of stimulatory cytokines (IL-15 or IL-2), bifunctional targeting (e.g., CD33/B16), and TCR-like CARs against neoantigens (e.g., NPM1 mutations), have further enhanced anti-leukemic potency and persistence.

Source selection and manufacturing approaches, ranging from peripheral blood and cord blood NK cells to iPSC-derived NK cells, play a critical role in balancing efficacy, scalability, and safety. PBMC-derived NK cells retain primary NK-cell biology and can provide potent cytotoxicity, but they are limited by donor-to-donor variability, finite expansion capacity, and variable transduction efficiency, which may affect product consistency and scalability [43, 70, 71]. Cord blood-derived NK cells provide an accessible allogeneic source and may be more amenable to banking, but they often require extensive ex vivo expansion and maturation, and their safety must be carefully evaluated in the context of myeloid antigen targeting [72, 73]. NK-92-based products offer high reproducibility and ease of genetic manipulation, but because NK-92 is a transformed cell line, clinical products require irradiation before infusion, which limits persistence and may reduce durable efficacy [74–76]. In contrast, iPSC-derived CAR-NK platforms, in particular, offer an off-the-shelf, multiplex-engineered solution capable of high-yield production and combinatorial modifications to improve persistence, cytotoxicity, and compatibility with antibody-based therapies [77–81]. However, iPSC-derived platforms also introduce additional translational challenges, including differentiation consistency, residual undifferentiated-cell testing, genomic stability assessment, release criteria, and regulatory complexity [80, 82]. Therefore, although iPSC-derived NK cells may offer the greatest scalability and engineering flexibility, primary NK-cell sources may remain attractive when preserving native effector function and minimizing stem cell-related regulatory concerns are prioritized. Collectively, these findings highlight the versatility and translational potential of CAR-NK therapies, supporting their advancement into clinical trials for AML and other myeloid malignancies. With continued optimization of CAR design, cytokine support, and gene-editing strategies, CAR-NK cells are poised to become a clinically viable, off-the-shelf immunotherapy platform capable of addressing the unmet needs in the treatment of myeloid cancers.

#### CAR-NKT cells

NKT cells are a specialized subset of αβ T cells distinguished by a limited TCR repertoire and a unique capacity to recognize glycolipid antigens. They exhibit characteristics akin to both conventional T cells and NK cells [83–85]. NKT cells are categorized into two primary subsets, termed type I and type II, based on their TCR repertoire. Type I NKT cells express semi-invariant TCR β chains in conjunction with an invariant TCR α chain (specifically, the Vα14 chain in mice and the Vα24 chain in humans), and are commonly referred to as invariant NKT (iNKT) cells [86]. These cells demonstrate a vigorous response to α-galactosylceramide (α-GalCer or αGC), a glycolipid derived from marine sponges [83–85]. In contrast, type II NKT cells possess a diverse, polyclonal TCR repertoire and are capable of recognizing lipid antigens such as sulfatide [87]. Research on type II NKT cells remains sparse, primarily due to their low abundance and the absence of well-defined surface markers [88].

Over the past five years, CAR-NKT cells have been developed and evaluated for their potent antitumor activity in both hematological malignancies and solid tumors, including multiple myeloma, B cell malignancies, AML, MDS, neuroblastoma, hepatocellular carcinoma (HCC), and renal cancer [18, 34, 89–99]. Notably, a recent clinical trial assessing GD2-targeting CAR-NKT cells for the treatment of pediatric patients with neuroblastoma demonstrated the safety and promising efficacy of this therapeutic approach, highlighting the potential of CAR-NKT cells in cancer treatment [100, 101].

CAR-NKT cells have been employed to target myeloid malignancies, including AML and MDS. A recent preclinical study successfully generated allogeneic CD33-targeting CAR-NKT cells from gene-engineered HSPCs using clinically guided culture methods (Fig. 2D) [18, 102]. These CAR-NKT cells can be produced with high yield, purity, and robustness, exhibiting several unique features that enhance their efficacy against bone marrow-resident AML and MDS.

First, these cells exhibit a predominant migratory ability toward the bone marrow, driven by their expression of specific chemokine receptors, particularly high levels of CXCR4 and CCR5 [18]. This property enables them to effectively target and eliminate bone marrow-resident blast cells. Second, the CAR-NKT cells demonstrate versatile tumor targeting capabilities, utilizing CAR, TCR, and natural killer receptor (NKR)-mediated mechanisms for killing. Notably, they can recognize CD33-low or CD33-negative LSCs in the bone marrow, which are typically resistant to conventional CD33-targeting CAR-T cells [18].

Additionally, NKT cells can effectively destroy CD1d^+^ tumor cells through their TCR, with this cytotoxicity further enhanced by HMAs, which significantly upregulate CD1d expression on AML tumor cells [18]. Indeed, these CAR-NKT cells have exhibited remarkable killing capacity both in vitro and in vivo, as demonstrated in various human AML xenograft mouse models and patient-derived xenograft (PDX) models [18].

Lastly, these allogeneic CAR-NKT cells show a favorable safety profile, characterized by the absence of GvHD, low incidence of CRS, minimal long-term toxicity, and reduced on-target, off-tumor effects against hematopoietic precursors [18, 103, 104]. Compared with CAR-NK cells, CAR-NKT cells may provide a distinct therapeutic advantage in myeloid malignancies because they combine innate-like cytotoxicity with TCR-dependent recognition of CD1d-presented lipid antigens [105, 106]. This multimodal recognition is particularly relevant in AML and MDS, where antigen heterogeneity and loss of CAR-targeted antigens can enable immune escape [107]. In the CD33-directed CAR-NKT platform, antileukemic activity was not limited to CAR recognition alone; CAR-NKT cells also recognized CD1d-positive leukemic cells through their endogenous invariant TCR and engaged tumor cells through natural killer receptor-mediated mechanisms [18]. This feature may allow CAR-NKT cells to eliminate CD33^low^ or CD33^−^ leukemia stem and progenitor populations that are less susceptible to conventional CD33 CAR-T cells.

Despite these advantages, CAR-NKT therapy remains less clinically mature than CAR-NK therapy in AML and MDS, as CD33-directed CAR-NK cells have already entered early-phase clinical evaluation in relapsed/refractory AML, whereas CAR-NKT studies in myeloid malignancies remain primarily preclinical [18, 60]. Key translational questions include whether CAR-NKT cells can persist long enough to eliminate residual disease without causing prolonged hematopoietic toxicity, whether repeated dosing will be required, and whether HSPC-derived manufacturing can be standardized across donors, gene-engineering designs, and differentiation batches [18]. In addition, the relative contribution of CAR-, TCR/CD1d-, and natural killer receptor-mediated killing may vary across patient samples depending on antigen density, CD1d expression, HMA exposure, and the composition of the bone marrow microenvironment [18, 103]. Therefore, future studies should define predictive biomarkers for CAR-NKT sensitivity considering these factors.

#### CAR-Ms

CAR-Ms hold considerable promise for cancer immunotherapy by reshaping the TME and eliciting a comprehensive antitumor immune response. Their intrinsic phagocytic activity and antigen-presenting capacity may enable more durable tumor control compared with other immune cell–based therapies. Through engineered CAR signaling, macrophages can be redirected to phagocytose opsonized or antigen-marked tumor cells [108–110]. For example, in HER2⁺ solid tumors (CT26 colon carcinoma and 4T1 triple-negative breast cancer) with limited response to anti-PD1 (aPD1) monotherapy, the combination of CAR-Ms with aPD1 has been shown in preclinical models to enhance tumor regression, prolong survival, and remodel the TME toward a more immunostimulatory state [111]. These findings highlight the potential synergy between CAR-M–based therapy and immune checkpoint blockade as a strategy to overcome resistance in otherwise non-responsive tumors.

For myeloid malignancies, macrophages may offer unique therapeutic advantages because they are native components of the bone marrow microenvironment where AML blasts reside and possess effector functions distinct from lymphocyte-based CAR platforms, including phagocytosis, cytokine secretion, antigen presentation, and remodeling of local immune niches [9, 112–114]. However, direct evidence supporting CAR-M therapy in AML or MDS remains limited, and most current CAR-M data come from solid tumor models [41, 110, 115, 116]. Therefore, the application of CAR-Ms to myeloid malignancies should be viewed as an emerging hypothesis rather than an established therapeutic strategy.

Several translational barriers are particularly important for CAR-M development. A key challenge will be the generation of macrophages with a stable M1-like phenotype, combining potent phagocytic and antigen-presenting properties, and maintaining consistent product quality, which includes optimizing the choice between autologous versus allogeneic sources, adenoviral versus lentiviral gene transfer, and macrophage generation from iPSCs or primary monocytes [108]. Macrophages also have limited proliferative capacity compared with lymphocytes, which may constrain in vivo expansion and require repeated dosing or local reprogramming strategies [117]. Manufacturing remains less standardized than for CAR-T or CAR-NK cell platforms, with unresolved questions regarding the optimal source cell, differentiation protocol, gene-transfer method, potency assay, and release criteria [117]. In addition, systemic activation of macrophages could produce inflammatory toxicity, particularly if CAR-Ms secrete pro-inflammatory cytokines or activate endogenous immune cells broadly [114, 115]. These issues are especially relevant in AML and MDS, where the therapeutic target tissue is the same compartment that supports normal hematopoiesis [112, 113].

Given the paucity of direct CAR-M studies in AML and MDS, this section necessarily places greater emphasis on mechanistic rationale, lessons from solid tumor CAR-M studies, and translational barriers rather than disease-specific efficacy data. We therefore interpret CAR-M therapy for myeloid malignancies as an emerging and hypothesis-generating platform, in contrast to CAR-NK therapy, which has more extensive AML-directed preclinical and early clinical evidence. Looking ahead, in vivo CAR engineering of monocytes and macrophages represents a highly attractive strategy to broaden the accessibility of CAR-M therapy [118–120]. Nanoparticle-based delivery systems, as well as viral and non-viral gene editing platforms, could enable direct reprogramming of macrophages within patients, bypassing ex vivo manufacturing hurdles. Such approaches may efficiently harness the intrinsic advantages of macrophages—tissue residency, phagocytosis, and antigen presentation—while providing precise tumor targeting. If successfully translated, in vivo CAR-M engineering could establish a versatile and scalable platform for treating both solid tumors and myeloid malignancies, with the potential for enhanced efficacy and improved safety.

### Clinical landscape of CAR cell therapies beyond CAR-T for treating myeloid malignancies

Currently, although no CAR-NKT or CAR-M therapies have been tested clinically in AML, several CAR-NK cell products have advanced to clinical evaluation (Table 2). The absence of CAR-NKT and CAR-M trials may be attributed to limited understanding of *their *in vivo persistence, expansion, and safety profiles in hematologic malignancies, as well as technical challenges associated with large-scale manufacturing and standardization of these cell types for clinical use. Although CAR-NKT and CAR-M therapies have not yet been clinically tested in AML or MDS, early clinical studies in other malignancies provide useful context for their translational feasibility. In a phase I trial of GD2-targeting CAR-NKT cells co-expressing IL-15 in children with relapsed or refractory neuroblastoma, treatment was clinically feasible and showed early evidence of antitumor activity, supporting the manufacturability and potential tolerability of CAR-NKT cells in humans (NCT03294954) [100, 101]. Similarly, CT-0508, an autologous HER2-targeting CAR-macrophage product, has entered clinical evaluation in patients with HER2-overexpressing solid tumors, providing initial human experience for CAR-M manufacturing, infusion safety, trafficking, and immune-modulatory activity (NCT04660929) [121, 122].

**Table 2:** Clinical investigation of CAR cell therapies beyond CAR-T for myeloid malignancies

CAR cell type	Disease indication	CAR target	Clinical design	Safety outcome	Efficacy outcome	ClinicalTrials.gov ID and ref
CAR-NK 92	Relapsed and refractory (R/R) AML	CD33	Patients received salvage chemotherapy, followed by infusion of CAR NK-92 cells irradiated with 60Co at 10 Gy	Infusions of CAR NK-92 cells, up to 5 × 10⁹ cells per patient, are safe and well-tolerated with no significant adverse effects	No clear clinical efficacy observed	NCT02944162 [123]
CAR-NK	R/R AML	CD33	Lymphodepletion with fludarabine (30 mg/m^2^) and cyclophosphamide (300–500 mg/m^2^) for 3 days, followed by one or more CAR-NK cell infusions	No ICANS or GVHD observed; no non-hematologic adverse events above grade 3 reported	With a median follow-up of 125 days (range 45–258), six of ten patients (60%) achieved MRD-negative complete response (CR), confirmed by imaging (iCR), at day 28	NCT05008575 [60]
CAR-NK	R/R AML	CD33/CLL1	CAR-NK cells given on days 1 & 3 of each 28-day cycle: 2 × 10^9^ cells first dose (cycle 1), 3 × 10^9^ cells thereafter if tolerated; all subsequent doses 3 × 10^9^ cells	Unknown	Unknown	NCT05215015
CAR-NK	R/R AML	CD123	Patients receive FC chemotherapy (F: Fludarabine; C: Cyclophosphamide) followed by CD123-CAR NK cell infusion	Unknown	Unknown	NCT05574608
CAR-NK	R/R AML	CD70	Dose-escalation study evaluating three dose levels of CAR-NK cells	Unknown	Unknown	NCT06696846
CAR-NK	R/R AML or blastic plasmacytoid dendritic cell neoplasm	CD123	Infusion of CAR-NK cells by dose of 1–10 × 10^6^ cells/kg	Unknown	Unknown	NCT06006403

Tang et al. reported the first-in-human trial of CD33-targeting CAR-NK-92 cells in three patients with relapsed or refractory AML (NCT02944162) [123]. In this study, NK-92-MI cells, engineered to express human IL-2 and transduced with a CD33-CAR construct, were infused at doses up to 5 × 10^9^ cells per patient. The treatment was well tolerated, with no substantial adverse events observed, although no clear clinical efficacy was reported [123].

Huang et al. generated CD33-targeting CAR-NK cells from umbilical cord-derived NK cells and evaluated their safety and efficacy in patients with relapsed or refractory AML (NCT05008575) [60]. Following a three-day lymphodepletion regimen with fludarabine (30 mg/m^2^) and cyclophosphamide (300–500 mg/m^2^), patients received one or more rounds of CAR-NK cell infusions. No immune effector cell–associated neurotoxicity syndrome (ICANS) or GvHD was observed, and adverse events greater than grade 3 were limited to hematological toxicity; no patients required intensive care unit admission. Regarding efficacy, with a median follow-up of 125 days (range 45–258 days), six of ten patients (60%) achieved minimal residual disease–negative complete response (MRD-CR) at day 28, as determined by imaging. This trial demonstrates the safety and preliminary efficacy of CD33 CAR-NK cells in AML, highlighting their potential to induce complete remission [60]. However, similar to CAR-T cell therapy, disease recurrence remains the primary cause of mortality, likely due to the limited in vivo expansion and persistence of CD33 CAR-NK cells, which resulted in long-term survival in only one patient [60].

Collectively, preclinical studies have established CAR-NK cells as a potent and versatile platform for targeting AML, demonstrating robust cytotoxicity, favorable safety profiles, and the ability to overcome key limitations of conventional CAR-T therapies, including reduced risk of GvHD and CRS. Early-phase clinical trials have now confirmed the safety of CD33-targeting CAR-NK cells derived from NK-92 or umbilical cord sources, with preliminary evidence of clinical activity, including MRD-negative complete remissions. These findings underscore the translational potential of CAR-NK therapy as an off-the-shelf treatment for AML. Nonetheless, several challenges remain before broader clinical adoption. Limited in vivo expansion and persistence of CAR-NK cells constrain long-term efficacy and durable responses, while recurrent disease continues to be a major cause of treatment failure. Continued optimization of manufacturing platforms and combinatorial treatment approaches will be critical to fully realize the clinical potential of CAR-NK therapy in AML. These advances may also pave the way for the eventual translation of CAR-NKT and CAR-M therapies, broadening the armamentarium of next-generation cellular immunotherapies for myeloid malignancies.

#### Limitations of current preclinical models

A major limitation across the preclinical literature is the heavy reliance on immunodeficient xenograft and PDX models. These systems are valuable for assessing direct antileukemic activity, tumor burden reduction, and preliminary safety; however, they incompletely model the human immune environment [124, 125]. In particular, NSG and related models lack fully functional adaptive and innate immune compartments, limiting their ability to predict host-versus-graft rejection, myeloid-mediated suppression, cytokine-driven toxicity, endogenous immune activation, and interactions between infused CAR cells and the human bone marrow microenvironment, which shaped the efficiency and persistence of CAR-NK, CAR-NKT, and CAR-M therapies [124, 126, 127]. Therefore, robust antileukemic activity in xenograft models should be interpreted as proof of concept rather than definitive evidence of clinical durability. The modest persistence and recurrent disease observed in early CAR-NK clinical studies highlight this translational gap and underscore the need for more predictive models [127, 128].

### Future development of CAR-NK, CAR-NKT and CAR-M therapies

#### Cytokine engineering strategy

Cytokine engineering represents a key strategy to enhance the efficacy, persistence, and in vivo expansion of next-generation CAR-based therapies, including CAR-NK, CAR-NKT, and CAR-M platforms. Unlike CAR-T cells, NK and NKT cells often exhibit limited longevity and proliferative capacity in vivo, which can restrict the durability of antitumor responses [58, 129–131]. Similarly, CAR-M therapies, while capable of potent tumor phagocytosis and modulation of the TME, may require additional support to sustain functional activity.

For CAR-NK cells, the ectopic expression of cytokines such as IL-15 or IL-2 has been shown to improve survival, expansion, and cytotoxic potency in both preclinical and early clinical studies. IL-15 expression can enhance CAR-NK persistence and resistance to immunosuppressive signals in the TME, although excessive cytokine production may contribute to systemic toxicity. IL-2 engineering, by contrast, may promote proliferation with a different safety profile, and optimization of dose, timing, and release kinetics is critical [50, 57, 58]. Combining cytokine engineering with checkpoint modulation, such as NKG2A disruption or co-stimulatory signaling (e.g., 2B4.ζ), can further enhance functional activity.

CAR-NKT cells similarly benefit from cytokine support, as IL-12, IL-15, IL-18, and IL-21 have been shown to drive expansion, promote a memory-like phenotype, and potentiate antitumor immunity [92, 98, 132–136]. Cytokine engineering in CAR-NKT cells may also help overcome the typically short lifespan of infused cells, improving in vivo persistence without inducing GvHD, a unique advantage over conventional T cells [52, 137–139].

For CAR-M therapies, cytokine engineering strategies are emerging to enhance macrophage survival, polarization toward a pro-inflammatory M1-like phenotype, and sustained phagocytic activity against tumor cells. Cytokines such as GM-CSF, IL-12, and IL-15 can be incorporated into CAR-M constructs or delivered in combination to enhance both direct tumor clearance and recruitment of endogenous immune cells [108, 110, 140, 141].

A central translational challenge is that cytokine engineering improves persistence and potency but may reduce controllability. For CAR-NK and CAR-NKT cells, IL-15 is attractive because it supports survival and expansion without requiring repeated exogenous cytokine administration. However, constitutive or soluble IL-15 expression can also drive excessive proliferation, systemic inflammation, or cytokine-associated toxicity, as shown in IL-15-secreting CD123 CAR-NK cells for AML and in studies comparing soluble versus membrane-bound IL-15 CAR-NK cell designs [50, 54, 142, 143]. IL-2 may enhance proliferation of activated T cells and NK cells, but it can also support regulatory T-cell expansion and has dose-dependent treatment-limiting toxicities, indicating a potentially narrower therapeutic window depending on dose and context [144–146]. IL-12 and IL-18 can promote inflammatory and memory-like effector programs, particularly when combined with IL-15 to generate cytokine-induced memory-like NK cells, but their systemic activity may raise additional safety concerns [147, 148]. Therefore, future cytokine-engineered products may require regulated expression systems, membrane-bound cytokines, local cytokine delivery, suicide switches, or pharmacologically controllable circuits to balance persistence with safety [149–151].

**Fig. 3:**
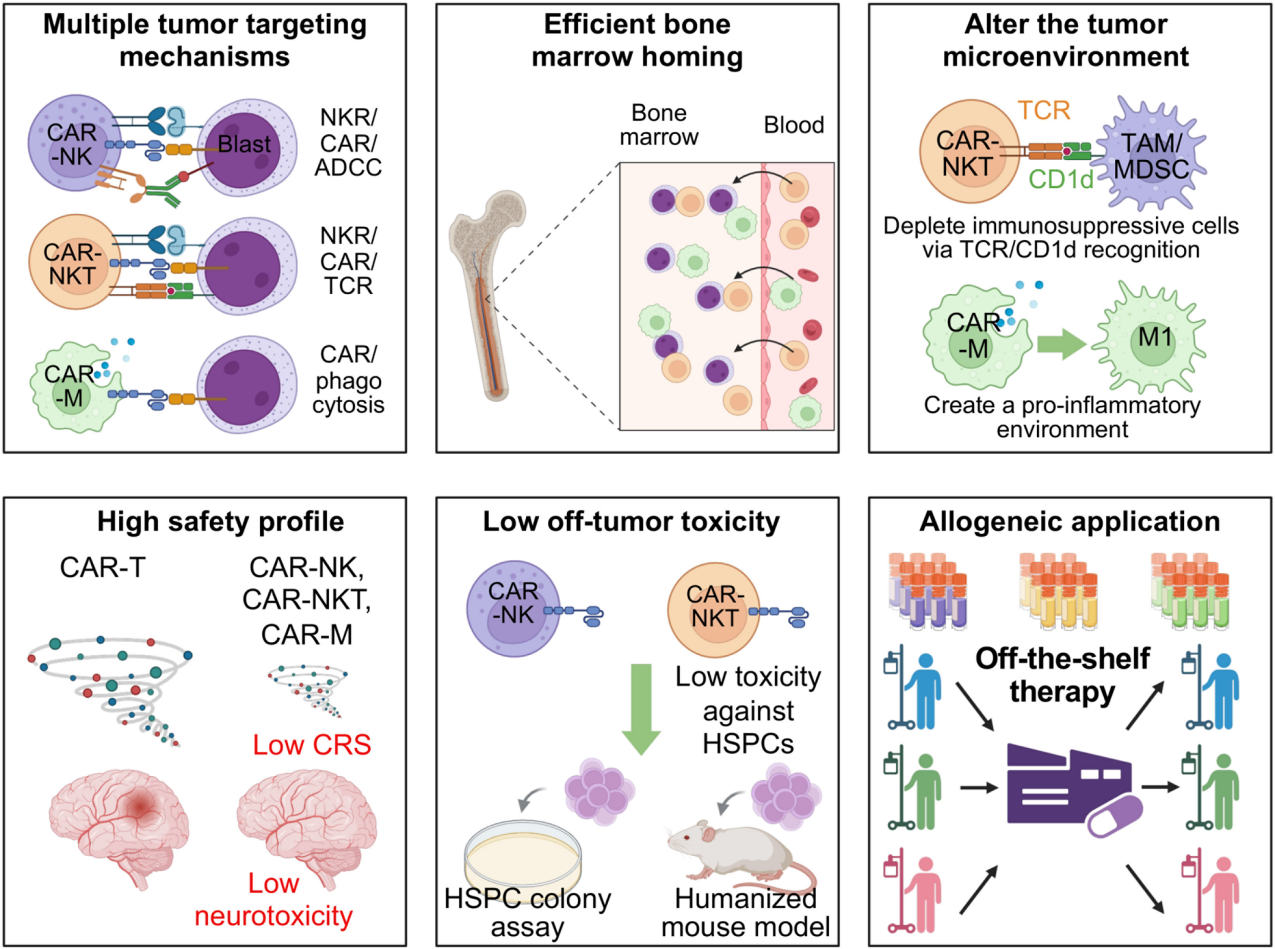
Therapeutic advantages of CAR-NK, CAR-NKT, and CAR-M cells in myeloid malignancies. CAR-NK, CAR-NKT, and CAR-M cell therapies offer several therapeutic advantages over conventional CAR-T approaches in myeloid malignancies. CAR-NK cells provide potent anti-leukemia activity while minimizing the risk of graft-versus-host disease and severe cytokine release syndrome due to their intrinsic innate-like cytotoxicity and short in vivo lifespan. CAR-NKT cells combine T cell–like specificity with NK-like innate functions, allowing rapid tumor targeting, cytokine-mediated immune modulation, and reduced risk of off-tumor toxicity, especially in the bone marrow microenvironment. CAR-M therapies can infiltrate the tumor niche efficiently, phagocytose leukemia cells, and remodel the immunosuppressive microenvironment, potentially overcoming leukemia stem cell–mediated relapse. Importantly, all three platforms can be developed as off-the-shelf allogeneic products, enabling rapid administration without patient-specific manufacturing. Collectively, these therapies offer enhanced safety, improved tumor targeting, and greater potential for durable remission in myeloid malignancies

Overall, cytokine engineering provides a versatile toolkit to improve the potency, persistence, and safety of CAR-NK, CAR-NKT, and CAR-M therapies. Future development will likely focus on optimized combinations of cytokine expression, CAR design, and cell-intrinsic modifications to generate next-generation “off-the-shelf” cellular therapies capable of durable, broad-spectrum antitumor activity (Fig. 3). These strategies have the potential to expand the applicability of CAR-based therapies to AML, other myeloid malignancies, and solid tumors while mitigating the limitations associated with conventional CAR-T cell approaches.

#### Combination therapy

Leveraging combination strategies with CAR-engineered cell therapies represents a critical future direction, as in many cases CAR cells alone may not be sufficient to fully overcome immune resistance or tumor immune evasion. Rational integration with agents such as checkpoint inhibitors, monoclonal antibodies, or oncolytic viruses can create synergistic effects that enhance antitumor efficacy. For example, in the context of CAR-NK cells, active combination approaches have included anti‐PD-1/PD‐L1 antibodies, cisplatin (chemotherapy), regorafenib (tyrosine kinase inhibitor), bortezomib (proteasome inhibitor), and oncolytic viruses engineered to express IL-15/IL-15Rα [152]. These combination strategies can augment CAR-NK function by enhancing persistence, increasing cytotoxicity, reversing the immunosuppressive tumor microenvironment, and promoting adaptive immune cross-priming. Similarly, combination therapy is likely to provide synergistic benefit for CAR-NKT cells and CAR-Ms. For CAR-NKT cells, co-administration with checkpoint blockade or cytokine support may further potentiate their dual ability to target tumor cells and reshape the tumor microenvironment through CD1d-restricted interactions. For CAR-Ms, combinations with checkpoint inhibitors, epigenetic modulators, or HMAs could enhance their ability to phagocytose tumor cells, present antigens, and recruit adaptive immunity [153–156].

Vaccine synergy has emerged as a promising strategy to enhance CAR-T cell therapy, with approaches including mRNA vaccines, peptide vaccines, viral vector vaccines, and dendritic cell (DC)-based vaccines [157]. These can be broadly categorized into two types: (i) vaccines that deliver antigens for presentation on major histocompatibility complex (MHC) molecules to activate native TCRs, and (ii) vaccines that directly stimulate CAR-T cells through CAR recognition [157]. In the context of other CAR-engineered immune cells, the first strategy may be particularly useful for CAR-NKT cells, where antigens presented on CD1d can activate the endogenous NKT TCR [84, 138, 158]. In contrast, this pathway is less relevant for CAR-NK and CAR-M cells, which lack TCR-mediated recognition. By comparison, the second strategy, which involves directly stimulating CAR cells via CAR recognition, is broadly applicable and can enhance the overall antitumor activity of CAR-engineered immune cells.

HMAs, such as azacitidine and decitabine, are DNA methyltransferase (DNMT) inhibitors that have been widely used in the treatment of myeloid malignancies, including AML and MDS [6, 159, 160]. Specifically, HMAs represent the frontline standard of care for higher-risk MDS patients and are also used in elderly or unfit AML patients who cannot tolerate intensive chemotherapy. In recent years, HMAs have been combined with the BCL-2 inhibitor venetoclax, establishing a new therapeutic standard. Despite their clinical utility, HMAs have several limitations: treatment responses often take months to emerge, the therapies are not curative, median response durations are limited, and resistance inevitably develops in most patients [6]. Importantly, mechanistic studies have revealed that HMAs may also potentiate immunotherapeutic strategies. A recent preclinical study demonstrated that HMAs upregulated the expression of several immune-related ligands on AML tumor cell line THP1, including the NKT TCR ligand CD1d and natural killer cell receptor ligands (CD112, CD155, MICA/B, and ULBPs) [18]. This phenotypic modulation rendered AML cells more susceptible to NKT cell–mediated cytotoxicity. Indeed, HMAs synergized with CD33-targeting CAR-NKT cells, and the combination therapy significantly enhanced tumor control in THP1 and KG1 xenograft mouse models, suggesting that epigenetic priming with HMAs can sensitize AML cells to CAR-NKT cell–mediated elimination and may represent a rational combination strategy [18].

In addition, clinical studies have demonstrated that HMA treatment can modulate target antigen expression on AML LSCs. In one study, azacitidine increased CD70 expression on LSCs in the peripheral blood of an AML patient after just one cycle of therapy, thereby amplifying CD70/CD27 signaling [160]. Disruption of this pathway using cusatuzumab, a human anti-CD70 monoclonal antibody with enhanced ADCC, effectively eliminated LSCs in vitro and in xenotransplantation experiments [160]. In a phase I clinical trial (NCT03030612), a single dose of cusatuzumab monotherapy followed by combination therapy with azacitidine demonstrated favorable safety and efficacy: among 12 enrolled patients, hematologic responses included 8 complete remissions, 2 complete remissions with incomplete blood count recovery, and 2 partial remissions, with 4 patients achieving minimal residual disease negativity (< 10^–3^ by flow cytometry) [160]. Taken together, these findings highlight the ability of HMAs to reprogram the immunophenotype of AML and MDS cells, thereby enhancing the effectiveness of immunotherapies. Combining HMAs with CD70-targeting CAR-based therapies may represent a particularly promising future direction to selectively eradicate LSCs, overcome resistance, and improve long-term outcomes for patients with myeloid malignancies.

##### Antigen selection and antigen escape

Antigen selection remains one of the most important determinants of safety and efficacy for CAR-based therapy in AML and MDS. Unlike B-cell malignancies, in which lineage-restricted antigens such as CD19 can be targeted with clinically manageable B-cell aplasia, many AML-associated antigens are also expressed on normal HSPCs or committed myeloid progenitors, creating a narrow therapeutic window for CAR-based targeting [161, 162]. CD33 and CD123 are among the most extensively studied targets, but both are associated with potential hematopoietic toxicity because of their expression on normal myeloid and hematopoietic progenitor populations [161, 163]. CLL-1 may provide improved selectivity because of its high expression on AML blasts and leukemia stem cells and its low or absent expression on normal HSPCs, although antigen expression may still vary across AML subclones and disease stages [164, 165]. Therefore, target selection must account not only for expression on bulk blasts, but also for expression on leukemia stem and progenitor cells, normal hematopoietic compartments, and relapse-initiating subclones [162].

On-target, off-tumor toxicity should also be evaluated in a platform-specific manner. CAR-T cells may pose the greatest risk of prolonged hematopoietic toxicity when directed against shared myeloid antigens because of their strong proliferative capacity and long-term persistence, particularly when targets such as CD33 or CD123 are also expressed on normal hematopoietic stem and progenitor cells [161, 166, 167]. CAR-NK cells may offer a more controllable toxicity profile because of their shorter persistence and lower risk of GvHD, but this does not eliminate hematopoietic risk. Indeed, the cord blood-derived CD33 CAR-NK cell study demonstrated reduced colony formation across multiple HSPC lineages in vitro, emphasizing that CAR-NK cells can still damage normal hematopoietic compartments when targeting antigens shared with HSPCs [60]. CAR-NKT cells may reduce toxicity through multimodal recognition and preferential bone marrow homing, but CD33-directed CAR-NKT strategies still require careful assessment of effects on normal myeloid progenitors [18]. CAR-M toxicity remains less defined in AML/MDS, but macrophage-mediated phagocytosis of normal antigen-positive hematopoietic cells and inflammatory remodeling of the marrow niche represent theoretical risks [41]. Thus, alternative CAR platforms may improve controllability or provide additional recognition mechanisms, but they do not solve the fundamental problem of shared antigen expression. Normal HSPC colony-forming assays, long-term hematopoietic reconstitution studies, cytokine profiling, and antigen-density threshold analyses should therefore be incorporated systematically during preclinical development [18, 60, 167].

Alternative CAR platforms may reduce but do not eliminate antigen escape. CAR-NK cells can kill through endogenous activating receptors when stress ligands are present, CAR-NKT cells can combine CAR specificity with CD1d/TCR- and natural killer receptor-mediated recognition, and CAR-Ms may promote antigen spreading through phagocytosis and antigen presentation. However, if the dominant leukemic population lacks the CAR-targeted antigen or downregulates antigen after therapy, all three platforms may lose efficacy. Accordingly, future strategies should include dual- or multi-antigen CARs, logic-gated recognition systems, combinatorial targeting of LSC-associated antigens, and epigenetic priming approaches that increase tumor immunogenicity [168]. In this context, HMAs are especially relevant because they are already used in AML and MDS and may increase expression of immune-recognition molecules, thereby sensitizing leukemic cells to CAR-NKT cell- or CAR-NK cell-mediated killing [18, 103, 104].

#### Allogeneic “off-the-shelf” application

Unlike conventional CAR-T cells, which require TCR knockout to mitigate GvHD, innate immune cells such as NK cells, NKT cells, and macrophages are inherently non-alloreactive and therefore do not induce GvHD [24, 28, 134, 169–171]. This unique property makes them particularly well-suited for allogeneic “off-the-shelf” applications. Importantly, TCR disruption in CAR-T cells has been associated with reduced persistence and impaired in vivo functionality, underscoring a major advantage of innate immune cells: they preserve their natural effector capacity without compromising antitumor efficacy [172–174].

The emergence of iPSC- and HSPC-derived CAR-NK, CAR-NKT, and CAR-M therapies further expands the potential of innate immune platforms by combining their inherent safety with the scalability and engineering flexibility of stem cell–based systems [37, 61, 62, 64, 102, 175–177]. One major strength of this approach is the ability to perform multiplex genetic engineering. For instance, targeted disruption of *B2M* or *CIITA* can ablate HLA class I and II molecules, thereby rendering engineered cells resistant to host T cell–mediated rejection [97, 99]. Simultaneously, enforced expression of non-classical HLA molecules such as HLA-E and HLA-G, or immune checkpoint molecules such as CD47, can protect these therapeutic cells from elimination by host NK cells and macrophages [178–181]. Additional edits, such as *NKG2A* knockout, have already been shown to enhance the antitumor activity of CAR-NK cells against AML, demonstrating how rational gene engineering can potentiate functional outcomes [49].

Another important advantage of stem cell–derived platforms is the ability to provide a standardized and inexhaustible supply of immune cells. Unlike donor-derived approaches, which are limited by variability in cell quality and availability, iPSCs can be clonally selected, genetically optimized, and expanded indefinitely. This allows for large-scale production of homogeneous, high-purity therapeutic products that can be manufactured in advance and stored, thus achieving the true vision of “off-the-shelf” allogeneic therapies. Such scalability not only supports clinical accessibility but also enables consistent batch-to-batch quality, which is critical for regulatory approval and widespread clinical translation [63, 77, 79, 182, 183].

Altogether, CAR-NK, CAR-NKT, and CAR-M therapies represent the next generation of allogeneic cell therapies. Their intrinsic lack of GvHD potential, combined with the scalability and engineering versatility of iPSC and HSPC platforms, offers significant advantages over conventional CAR-T cells. Future development is likely to focus on extending in vivo persistence, improving tumor trafficking and infiltration, reinforcing resistance to immunosuppression, integrating rational safety mechanisms, and reducing host cell-mediated allorejection [86, 108, 184, 185]. With these innovations, innate immune cell–based CAR therapies are poised to become a cornerstone of off-the-shelf immuno-oncology and may ultimately broaden the reach of cell therapy to a wider spectrum of cancers, including myeloid malignancies.

In the context of myeloid malignancies, the use of allogeneic CAR-engineered cells offers distinct advantages over autologous CAR-T cell approaches. A major challenge in targeting myeloid leukemias is the risk of on-target, off-tumor cytotoxicity, since most candidate antigens (e.g., CD33 and CD123) are also expressed on normal HSPCs. This raises significant safety concerns when using long-lived autologous CAR-T cells, which can cause prolonged hematopoietic toxicity [15, 21, 186]. In contrast, allogeneic CAR-NK, CAR-NKT, and CAR-M therapies provide a unique therapeutic window: they can effectively eliminate malignant blasts but are ultimately cleared by the host immune system, thereby mitigating the long-term depletion of normal HSPCs [18, 98]. This natural rejection not only enhances the safety profile but also distinguishes them from autologous CAR-T cells, which may persist indefinitely and sustain toxicity. Moreover, the allogeneic nature of these therapies allows for repeat dosing once the infused cells are rejected, enabling a controlled and flexible treatment strategy. Taken together, these properties highlight an additional layer of benefit conferred by allogeneic innate immune cell–based CAR therapies in the treatment of myeloid malignancies, where long-term safety remains a critical unmet need.

#### In vivo CAR engineering

In vivo CAR engineering refers to the direct genetic modification of endogenous immune cells within the patient’s body, bypassing the need for ex vivo cell isolation, expansion, and reinfusion. This is typically achieved through nanoparticle-mediated or viral vector–based delivery systems that transport CAR-encoding nucleic acids to specific immune cell populations [118]. Compared with NK and NKT cells, macrophages are particularly attractive targets for in vivo CAR engineering. Macrophages constitute a large proportion of circulating immune cells as well as tissue-resident populations in organs such as the bone marrow, liver, and spleen. Moreover, their intrinsic phagocytic capacity makes them highly amenable to uptake of lipid nanoparticles (LNPs) or other nanoparticle-based delivery systems, providing a natural advantage for efficient in vivo transduction [118].

Several preclinical studies have demonstrated proof-of-concept for this approach. For example, intravenous administration of LNPs containing plasmid DNA encoding a MUC1-targeted CAR successfully reprogrammed intratumoral macrophages, driving potent antitumor immunity against pancreatic adenocarcinoma [187]. Similarly, LNPs carrying mRNA encoding a GPC3-specific CAR in combination with Siglec-G lacking inhibitory ITIMs were intravenously delivered to generate CAR-macrophages that activated both innate and adaptive immune responses against hepatocellular carcinoma [188]. In another study, intratumoral injection of a nanoporter–hydrogel superstructure carrying plasmid DNA for a CD133-specific CAR generated glioblastoma stem cell (GSC)–targeting CAR-macrophages, effectively clearing residual tumor cells and preventing postoperative recurrence [189]. Collectively, these studies underscore the potential of in vivo CAR engineering to harness macrophages as therapeutic effectors against solid tumors.

Extending this strategy to myeloid malignancies is particularly compelling, given macrophages’ natural residency and trafficking within the bone marrow microenvironment, where leukemic blasts reside. However, several challenges must be addressed before clinical translation. These include identifying highly specific surface targets on leukemic cells to ensure selective CAR-mediated recognition while sparing normal hematopoiesis, optimizing delivery platforms to direct CAR constructs preferentially to bone marrow macrophages while minimizing off-target transduction of tissue-resident macrophages in vital organs such as the liver and lungs, and developing safety switches to mitigate potential risks of excessive inflammation or cytokine-driven toxicity [118, 190]. Furthermore, improving the durability of in vivo–engineered CAR expression, standardizing dosing strategies, and assessing long-term immune memory induction remain key areas for future investigation. Overall, in vivo CAR engineering of macrophages offers a promising and scalable approach to safely and effectively target myeloid malignancies, but its success will depend on overcoming these technical and biological hurdles.

#### Manufacturing, scalability, and regulatory considerations

Although off-the-shelf CAR-NK, CAR-NKT, and CAR-M therapies may improve accessibility compared with autologous CAR-T therapy, each platform introduces distinct manufacturing and regulatory challenges [29, 191–193]. For CAR-NK therapies, the selection of PBMC-, cord blood-, NK-92-, or iPSC-derived starting material introduces source-specific tradeoffs in scalability, persistence, safety testing, and product standardization, as discussed above [191]. Stem cell-derived platforms, including iPSC- and HSPC-derived CAR products, may address some donor-variability constraints by enabling clonal master cell banks, multiplex gene engineering, and large-scale production, but they require rigorous evaluation of genomic stability, differentiation fidelity, residual undifferentiated cells, insertional mutagenesis risk, batch-to-batch consistency, and long-term safety. CAR-M products introduce additional challenges because macrophages are highly plastic and functionally heterogeneous, making it necessary to define potency assays that capture not only CAR-dependent target engagement but also antigen-specific phagocytosis, inflammatory polarization, cytokine secretion, antigen presentation, and T cell recruitment.

Regulatory expectations will also differ across platforms. For multiplex-engineered iPSC-derived products, release testing must demonstrate identity, purity, viability, sterility, potency, karyotypic/genomic stability, and absence of residual pluripotent cells [194]. Regarding HSPC-derived CAR-NKT cell products, standardization of differentiation efficiency, NKT cell purity, CAR expression, alloreactivity testing, and functional potency will be essential to ensure batch consistency across donor sources and engineering designs. In terms of in vivo CAR engineering, additional concerns include biodistribution of the delivery vehicle, off-target transduction, duration of CAR expression, dose control, reversibility, and management of inflammatory toxicity [118, 195, 196]. These issues highlight that clinical translation will depend not only on antitumor activity, but also on robust chemistry, manufacturing, and controls strategies tailored to the biology of each CAR-engineered immune cell platform.

## Discussion

CAR-engineered immune cell therapies have transformed the treatment landscape of hematologic malignancies, yet their application in myeloid malignancies such as AML and MDS remains limited. Conventional CAR-T cell therapies face significant challenges in this context, including poor bone marrow trafficking, limited activity against leukemia stem cells, antigen overlap with normal HSPCs, and high risks of on-target, off-tumor toxicity. Additionally, autologous CAR-T therapies are constrained by manufacturing complexities, patient variability, and the potential for GvHD in allogeneic settings. These limitations underscore the urgent need for next-generation CAR platforms that combine potent anti-leukemic activity with improved safety, persistence, and broad accessibility (Fig. 1).

The three alternative CAR platforms discussed in this review address CAR-T limitations through distinct biological mechanisms (Figs. 2 and 3). CAR-NK cells primarily provide an immediately cytotoxic, off-the-shelf effector platform with reduced GvHD risk and the ability to kill through both CAR-dependent mechanisms and CAR-independent activating receptors [197, 198]. Their main translational limitation is insufficient in vivo persistence, which may explain why early clinical responses can be transient despite favorable safety, as observed in early-phase CD33 CAR-NK studies in relapsed/refractory AML [60, 127]. CAR-NKT cells offer a different balance of properties that they retain cytotoxic capacity but also possess tissue-homing and immunoregulatory features, and their endogenous invariant TCR enables CD1d-dependent recognition that may complement CAR targeting [18, 98]. This trait may be particularly valuable in AML and MDS, where leukemia stem and progenitor cells can display heterogeneous or low expression of myeloid CAR antigens. CAR-Ms are the least clinically mature platform in myeloid malignancies, but they provide functions not shared by CAR-NK or CAR-NKT cells, including direct phagocytosis, antigen presentation, and local remodeling of immune niches [41, 110, 114]. Therefore, these platforms should be selected according to the dominant therapeutic problem, including rapid cytoreduction may favor CAR-NK cells, bone marrow homing and multimodal recognition may favor CAR-NKT cells, and microenvironmental remodeling or antigen spreading may favor CAR-M-based strategies.

Cytokine engineering and combination therapy strategies further expand the potential of these next-generation CAR therapies. Ectopic expression of cytokines such as IL-15, IL-12, and IL-18 can enhance persistence, functional activity, and expansion of CAR-NK, CAR-NKT, and CAR-M cells, while combination with HMAs, checkpoint inhibitors, or vaccines may improve tumor targeting and overcome immune evasion [19, 20, 199]. Preclinical data indicate that epigenetic priming with HMAs can upregulate tumor antigen expression, thereby sensitizing AML cells to CAR-mediated killing and providing a rational approach to eradicate LSCs [18].

The development of stem cell–derived platforms, including iPSC- and HSPC-based CAR therapies, represents a transformative advance, enabling scalable, standardized, and multiplex-engineered “off-the-shelf” products. These approaches allow for the incorporation of multiple genetic modifications to improve persistence, cytotoxicity, immune evasion, and safety while providing a reproducible supply of high-quality therapeutic cells. In vivo CAR engineering, particularly of macrophages, holds additional promise as a scalable and minimally invasive strategy to target bone marrow-resident malignant cells while harnessing intrinsic tissue-resident properties.

Despite these advances, several challenges remain before widespread clinical adoption. Optimizing in vivo persistence, controlling cytokine-mediated toxicity, enhancing tumor infiltration, and ensuring selective targeting of malignant cells while sparing normal hematopoietic populations are critical areas for future investigation. Furthermore, large-scale manufacturing, regulatory standardization, and cost-effective delivery strategies will be essential to translate these therapies into routine clinical practice.

In conclusion, CAR-NK, CAR-NKT, and CAR-M therapies represent the next frontier in cellular immunotherapy for myeloid malignancies. By integrating innate immune cell biology with advanced genetic engineering, cytokine modulation, and combinatorial strategies, these platforms have the potential to overcome the limitations of conventional CAR-T cell therapy, providing safe, effective, and broadly accessible treatments. Continued preclinical optimization and early-phase clinical evaluation will be crucial to realizing their promise, with the ultimate goal of improving outcomes for patients with AML, MDS, and other difficult-to-treat myeloid cancers.

## Data Availability

No datasets were generated or analysed during the current study.

## Abbreviations

ADC: Antibody–drug conjugate
ADCC: Antibody-dependent cellular cytotoxicity
allo-HSCT: Allogeneic stem cell transplantation
AML: Acute myeloid leukemia
BM: Bone marrow
CAR: Chimeric antigen receptor
CAR-NK: CAR-natural killer
CAR-NKT: CAR-invariant natural killer T
CAR-M: CAR-macrophage
CIML: Cytokine-induced memory-like
CLL1: C-type lectin-like molecule-1
CRS: Cytokine release syndrome
FLT3: Fms-like tyrosine kinase 3
GvHD: Graft-versus-host disease
HCC: Hepatocellular carcinoma
HMA: Hypomethylating agent
HSPC: Hematopoietic stem and progenitor cell
LNP: Lipid nanoparticle
LSC: Leukemia stem cell
MDS: Myelodysplastic syndrome
MM: Multiple myeloma
NKR: Natural killer receptor
TME: Tumor microenvironment
UTD: Untransduced
